# Preliminary exploration of the effects of environmental factors on the microsatellite status of BRAF-mutated colorectal cancer

**DOI:** 10.1186/s12957-023-03106-6

**Published:** 2023-08-25

**Authors:** Binle Tian, Guiming Chen, Xiaoqin Shi, Liren Jiang, Tao Jiang, Qi Li, Lin Yuan, Jian Qin

**Affiliations:** 1 Department of Oncology, Shanghai General Hospital, Shanghai Jiao Tong University School of Medicine, 100 Haining Road, Shanghai, 200080 China; 2 Department of General Surgery, Shanghai General Hospital, Shanghai Jiao Tong University School of Medicine, 100 Haining Road, Shanghai, 200080 China; 3 Pathology Center, Shanghai General Hospital, Shanghai Jiao Tong University School of Medicine, 100 Haining Road, Shanghai, 200080 China

**Keywords:** Colorectal cancer, BRAF, Microsatellite status, EBV, Gallstone disease

## Abstract

**Background:**

To investigate the expression of EBV products and frequency of gallstone disease (GD) among different microsatellite status in colorectal cancer (CRC) with BRAF^V600E^ mutation.

**Methods:**

We collected 30 CRC patients with BRAF^V600E^ mutation and 10 BRAF ( −) CRC patients as well as 54 healthy subjects. Tumor tissue samples were collected to detect the mutation of BRAF, KRAS, and TP53. Microsatellite status was determined by immunohistochemistry and PCR. EBER in situ hybridization was performed to detect EBV. In addition, we also collected clinical information about the patients.

**Results:**

We found that although EBV products were detected in CRC, there were no significant differences in the EBV distribution between the different BRAF groups. Our study demonstrated that BRAF^V600E^ mutation and BRAF^V600E^ with MSI were significantly more frequent in the right CRC. Furthermore, the KRAS mutation rate in the BRAF-wild-type group was proved to be significantly higher than that in the BRAF mutation group. In addition, we revealed that BRAF mutation and MSI were independent risk factors of TNM stage. The frequency of GD was higher in CRC patients than in general population, and although there was no significant difference between CRC with or without BRAF^V600E^ mutation, the highest frequency of GD was found in MSS CRC with BRAF^V600E^ mutation.

**Conclusions:**

EBV plays a role in CRC, but is not a determinant of different microsatellite status in CRC with BRAF^V600E^ mutation. The frequency of GD in MSS CRC with BRAF^V600E^ mutation is significantly higher than that in the general population.

## Introduction

Most of the colorectal cancer (CRC) patients with V-raf murine sarcoma viral oncogene homolog (BRAF) V600E mutations are derived from sessile serrated adenoma (SSAD) of the proximal colon, while a fraction of them is derived from traditional serrated adenoma (TSA) of distal colon with special molecular and clinicopathological features. BRAF^V600E^ mutations of CRC are mostly characterized by high levels of CpG island methylator phenotype (CIMP) of the gene promoter, mainly caused by mismatch repair gene MLH-1 promoter methylation and microsatellite instability (MSI) as a result of gene silencing, which is considered an early event in the SSAD-carcinoma transformation [1]. The remaining approximate 25% of CRC patients carrying BRAF^V600E^ mutation showed a high level of CDKN2A (p16) promoter methylation along with a high level of CIMP without MLH-1 promoter methylation indicating microsatellite stability (MSS) [2]. There was no difference in the survival rate of CRC patients carrying BRAF^V600E^ mutation with MSI-H when compared to those with unmutated BRAF; however, BRAF^V600E^ mutated CRC with MSS was highly malignant and lack of effective treatment, resulting in a very poor prognosis [3, 4].

There are few studies on the mechanisms of methylation of MSS CRC with BRAF^V600E^ mutation. The molecular characteristics of MSS CRC with BRAF^V600E^ mutation are highly similar to Epstein-Barr virus-associated gastric cancer (EBVaGC) [5]. EBV-positive tumors have a high prevalence of DNA hypermethylation, and almost all EBVaGCs display CDKN2A (p16INK4A) promoter hypermethylation without MLH1 hypermethylation characteristic of MSI-associated CIMP, exhibiting MSS [6]. Thus, we speculate that there may be some correlation between MSS CRC with BRAF^V600E^ mutation and EBV infection.

Study reports of EBV-associated CRC are almost none; thus, the role of EBV in CRC is still uncertain. In general, EBV plays a role in the pathogenesis of colon cancer, and a chronic inflammatory environment can facilitate the carcinogenicity of EBV [7]. However, most of the existing researches have some defects in the study design, such as inappropriate sample properties and sample size, which lead to the inconclusive role of EBV in the development of CRC.

It is especially noteworthy that many of the risk factors for gallstone disease (GD) include factors that are well-established as risk factors for CRC (obesity, high-energy intake, alcohol consumption, and diabetes) [8]. Bile acids (BAs), particularly secondary BAs, can cause oxidative stress, DNA damage, apoptosis, and mutation and were speculated to be strong carcinogens or promoters of CRC [9, 10].

The aim of this study was to compare the expression of EBV products and the frequency of GD among different microsatellite status in CRC with BRAF^V600E^ mutation and to investigate the role of EBV and GD in the pathogenesis of MSS CRC with BRAF^V600E^ mutation.

## Materials and methods

### Patient selection and patient characteristics

From January 2018 to December 2020, 30 CRC patients with BRAF^V600E^ mutation and 10 BRAF ( −) CRC patients after surgical resection as well as 54 healthy subjects were collected from Shanghai General Hospital. In addition to the collection of tissue specimens, we also collected clinical information about the patients, especially the gallstone situation. All CRC patients underwent preoperative hepatobiliary ultrasound and upper abdominal-enhanced CT scan. And all healthy subjects were community routine physical examination population and received B-ultrasound examination of liver, biliary, and pancreas. The diagnosis of gallstones relies on imaging findings or a history of cholecystectomy for gallstones. Our research was approved by the Ethics Committee for Clinical Research of Shanghai General Hospital and was conducted in accordance with the Declaration of Helsinki.

### Detection of BRAFV^600E^ mutation

The mutations of BRAF^V600E^ were detected by qPCR. The nucleic acid was extracted by a QIAamp DNA Mini Kit (Kaijie Biological Engineering Co., Ltd.), and OD260/OD280 was used for quality control. BRAF Exon 15-specific primer (forward primer 5′-TCATAATGCTTGCTGTGATAGGA-3′and reverse primer 5′-GGCCAAAAATTTAATCAGTGGA-3′) was designed, and real-time PCR was performed and analyzed by ABI 3730 DNA sequencer.

### Determination of MSI

The total genomic DNA was extracted by a QIAmp DNA Mini Kit, and OD260/OD280 was used for quality control. The microsatellite genomic DNA was detected by Promega multiplex quantitative PCR. Five single-nucleotide sites (BAT-25, BAT-26, Mono-27, NR-21, Nr-24) and two control sites (PentaC, PentaD) were used as the reference for MSI analysis. All five stable sites indicated MSS, one unstable site indicated low-degree microsatellite instability (MSI-L), and two or more unstable sites indicated high-degree microsatellite instability (MSI-H). The tissues were also fixed with paraformaldehyde and embedded in paraffin for section staining. Four MMR proteins (MLH1, PMS2, MSH2, and MSH6) were stained by immunohistochemistry. High expression of the four MMR proteins was considered as MSS. The absence of expression of any MMR proteins indicates that the tissue is MSI.

### Detection of EBV

EBER in situ hybridization was performed according to the instructions of EBER in situ hybridization kit. The procedure was as follows: first, 4-µm-thick paraffin-embedded tissue sections were dewaxed and hydrated by xylene and gradient alcohol; second, protease digestion; third, digoxin-labeled EBER probe hybridization; then, incubation with HRP-labeled anti-digoxin antibody; and finally, DAB chromogenic. EBV positivity was defined as 20% or more of EBER expression in tumor cells. Staining results of all tissue sections were independently interpreted by two full-time pathologists.

### Detection of KRAS

After total genomic DNA isolation, in vitro amplification of codons 12 and 13 of proto-oncogene KRAS was carried out in a total volume of 25-μl reaction mixture containing 15-μl amplification mixed with biotin-labeled primers, 4.5-μl Taq dilution buffer, 2.5-U Taq polymerase, and 50–100-ng genomic DNA. PCR condition of KRAS codons 12 and 13 mutations is as follows: initial denaturation step of 94 °C for 15 min, followed by 35 cycles of denaturation at 94 °C for 1 min, annealing at 70 °C for 30 s and 58 °C 50 s, elongation at 72 °C for 1 min, and a final elongation at 72 °C for 7 min. To detect KRAS codons 12 and 13 mutations, we modified the artificial RFLP/PCR approach used by previous investigators [11].

### Detection of TP53

PCR single‐stranded conformation polymorphism (PCR‐SSCP) was applied to identify the TP53 mutation in exons 5–8. The PCR products (2 μL) were mixed with 10 μL of gel loading solution (9.5% deionized formamide, 20-mM EDTA‐Na, 0.05% xylene cyanol and bromphenol blue) and then denatured at 95 °C for 5 min. Nondenaturing 7.5% polyacrylamide gels were used for electrophoresis at 260–300 V for 3–12 h, with the temperature controlled at 22 °C using a temperature controller. The gels were visualized by silver staining and photographed. The migrated band was removed from the gel, and the DNA was extracted. Suspected mutations obtained by SSCP in the TP53 gene were then confirmed by sequence analysis.

### Statistical analysis

Continuous data are expressed as arithmetic mean and standard deviation (SD) or median and range. Student’s two-sided *t*-test was used to compare the means of the two groups. All categorical data are presented as absolute and relative frequencies and compared to controls using the chi-square test (Fisher’s exact test, Pearson’s chi-squared test, or continuity-modified chi-square test). A *p*-value < 0.05 was defined as significant for all other statistical tests. Calculations were performed using GraphPad Prism (version 8.0.1).

## Results

### Patient characteristics and EBV distribution

**Fig. 1:**
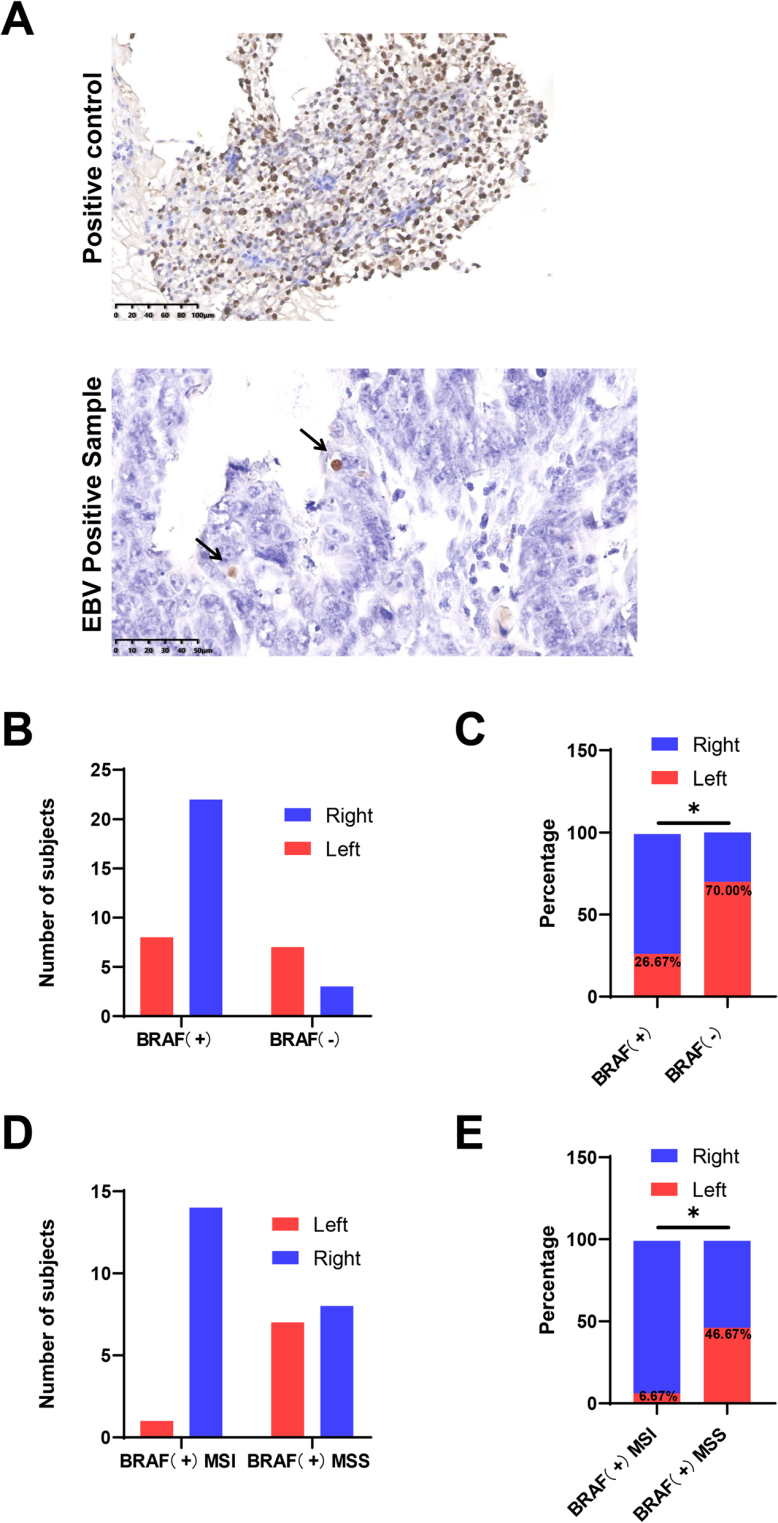
**A** The expression of EBV in CRC tissues was detected by EBER in situ hybridization. Scale bars: 100 µm and 50 µm. **B** and **C** The location of CRC tumors with or without BRAF mutation. **D** and **E** The location of BRAF^V600E^ with MSI CRC tumors or BRAF^V600E^ with MSS CRC tumors. *p < 0.05

Characteristics of all 40 included patients are described in Table 1. Most of the patients were men (62.5%). Fifteen tumors were in the left colon (37.5%) and 25 in the right colon (62.5%). The age of the BRAF-positive MSI group (70.67 ± 8.83) was older than that of the BRAF-positive MSS group (68.07 ± 10.42) and the BRAF-wild-type group (66.60 ± 9.38). The expression of EBV in CRC tissues was detected by EBER in situ hybridization (Fig. 1A). Five patients were EBV positive (12.5%), and 35 patients were EBV negative (87.5%). However, there were no significant differences in the EBV distribution between the different BRAF groups. The results showed that BRAF^V600E^ mutation was significantly more frequent in the right CRC (*p* = 0.0237, Fig. 1B, C). Furthermore, BRAF^V600E^ with MSI CRC was also significantly more frequent in the right colon compared with BRAF^V600E^ with MSS CRC (*p* = 0.0352, Fig. 1D, E).

**Table 1:** Patient characteristics and EBV distribution

	Gender		Location		Age (years)			EBV	
	Male	Female	Left	Right	< 60	≥ 60	Mean ± SD	Positive	Negative
BRAF + MSI	10	5	1	14	2	13	70.67 ± 8.83	2	13
BRAF + MSS	8	7	7	8	3	12	68.07 ± 10.42	2	13
BRAF ( −)	7	3	7	3	2	8	66.60 ± 9.38	1	9

### BRAF and KRAS mutations in CRC patients

**Fig. 2:**
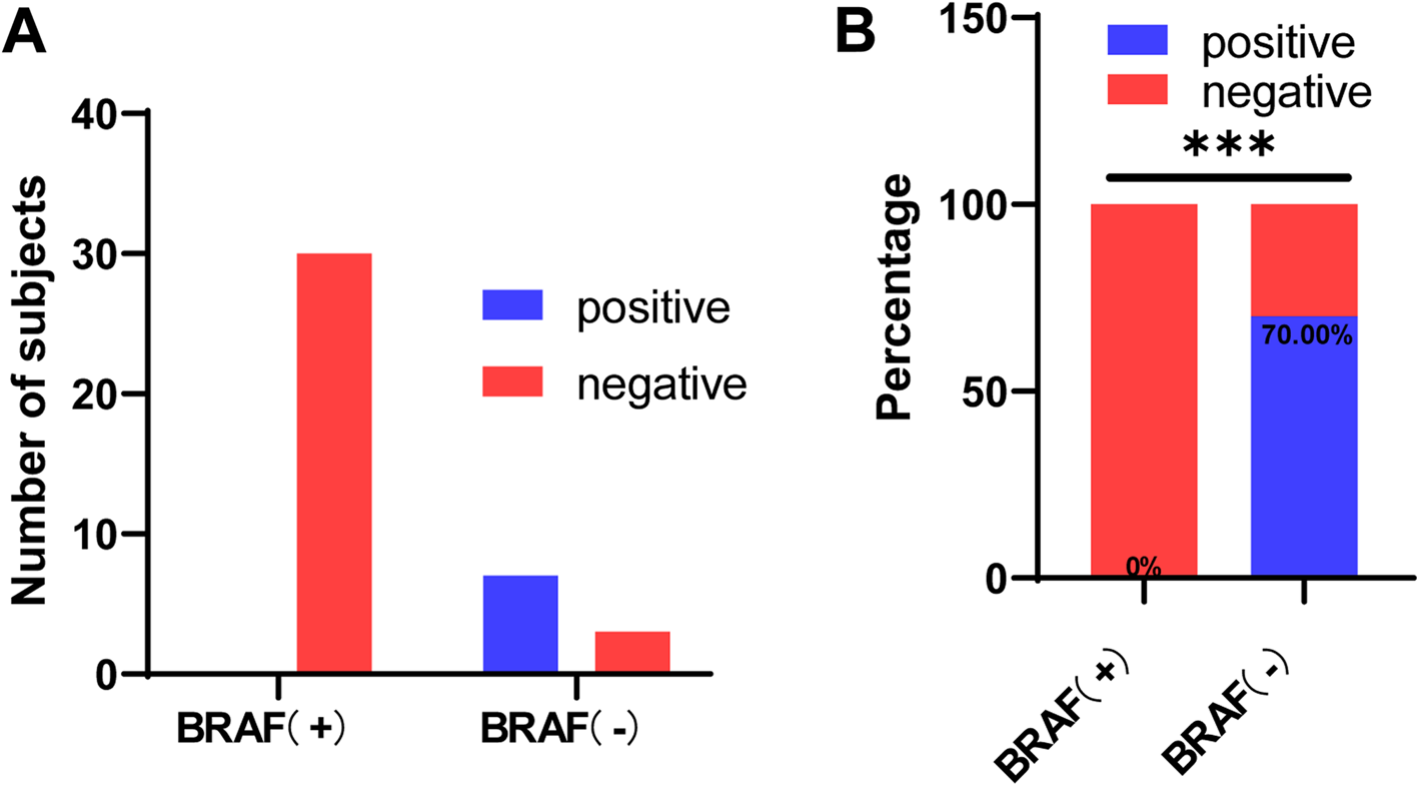
**A**, **B** The KRAS mutation of CRC tumors with or without BRAF mutation. ***P < 0.001

As shown in Table 2, all KRAS in BRAF-positive group were wild type. There were 10 cases in BRAF-wild-type group, including 3 wild-type cases and 7 KRAS mutation cases. The KRAS mutation rate in the BRAF-wild-type group was significantly higher than that in the BRAF-positive group (*p* < 0.0001, Fig. 2A, B).

**Table 2:** BRAF and KRAS mutation in CRC patients

	KRAS mutation	
	( +)	( −)
BRAF ( +)	0	30
BRAF ( −)	7	3

### BRAF and TP53 mutations in CRC patients

Most data about TP53 were lost. In BRAF-positive MSI group, 6 cases had complete data, among which 1 case was TP53 mutation positive, 3 cases had a minor expression of TP53 mutation, and 2 cases had no TP53 mutation. In the BRAF-positive MSS group, 3 patients with complete data were all TP53 mutation positive, and all located in the right colon. The difference between the left and right colon was probably related to the excessive lack of this data in this study. The cases in BRAF-wild-type group were too fewer and incomplete to perform any comparative analysis.

### BRAF mutation and TNM stages of CRC patients

**Fig. 3:**
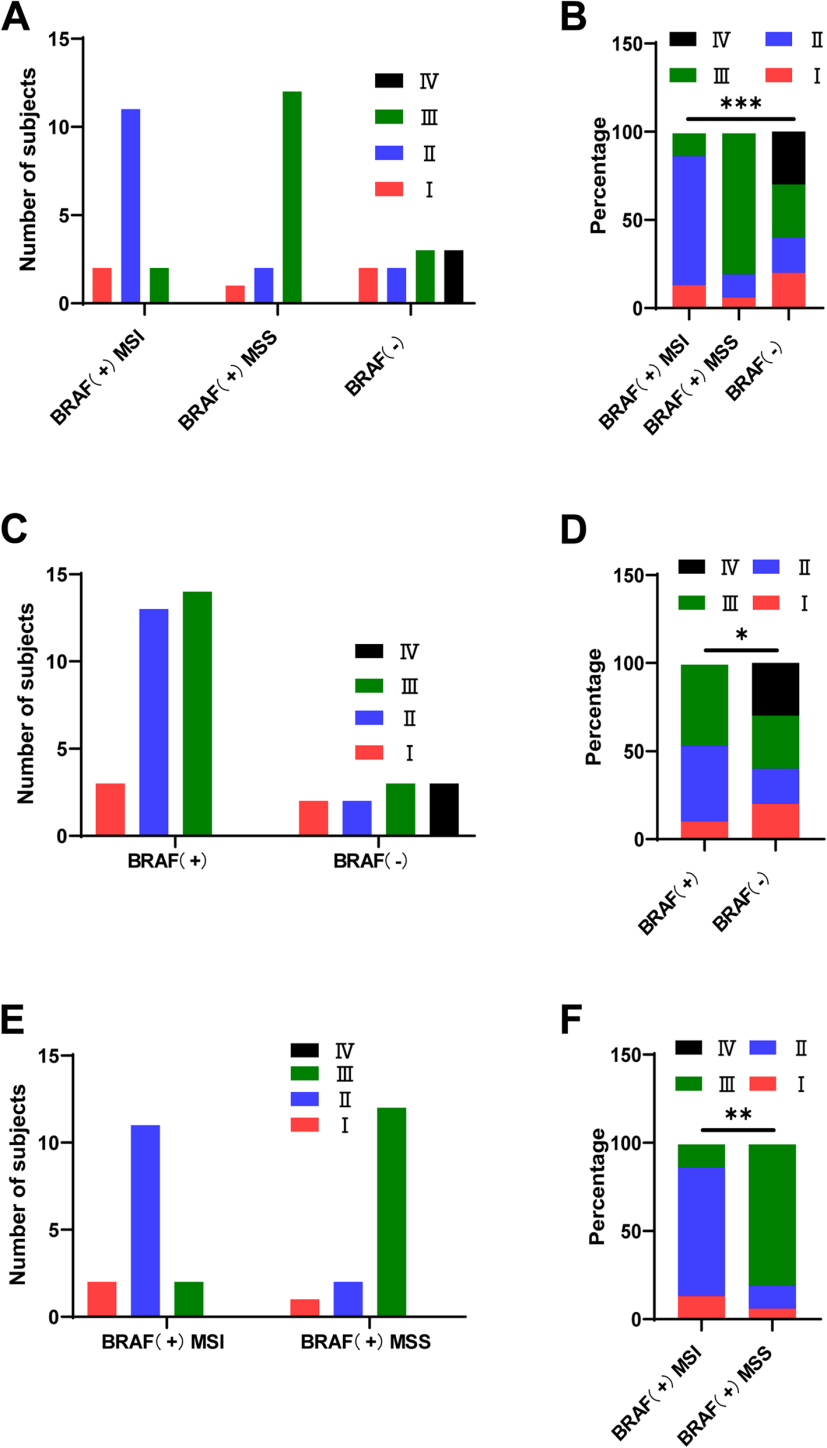
**A**, **B** The TNM stages of CRC patients in different groups. **C**, **D** The TNM stages of CRC patients with or without BRAF mutation. **E**, **F** The TNM stages of BRAF^V600E^ with MSI CRC tumors or BRAF^V600E^ with MSS CRC patients. *P < 0.05, **P < 0.01, ***P < 0.001

Our results shown in Table 3 suggested that the TNM stages of patients in different groups were significantly different (*p* = 0.0002, Fig. 3A, B). Further analysis revealed that BRAF mutation (*p* = 0.0108, Fig. 3C, D) and MSI (*p* = 0.0011, Fig. 3E, F) were independent risk factors of TNM stages.

**Table 3:** BRAF mutation and TNM in CRC patients

	TNM stage (UICC)			
	I	II	III	IV
BRAF + MSI	2	11	2	0
BRAF + MSS	1	2	12	0
BRAF ( −)	2	2	3	3

### BRAF mutation and gallstone in CRC patients

**Fig. 4:**
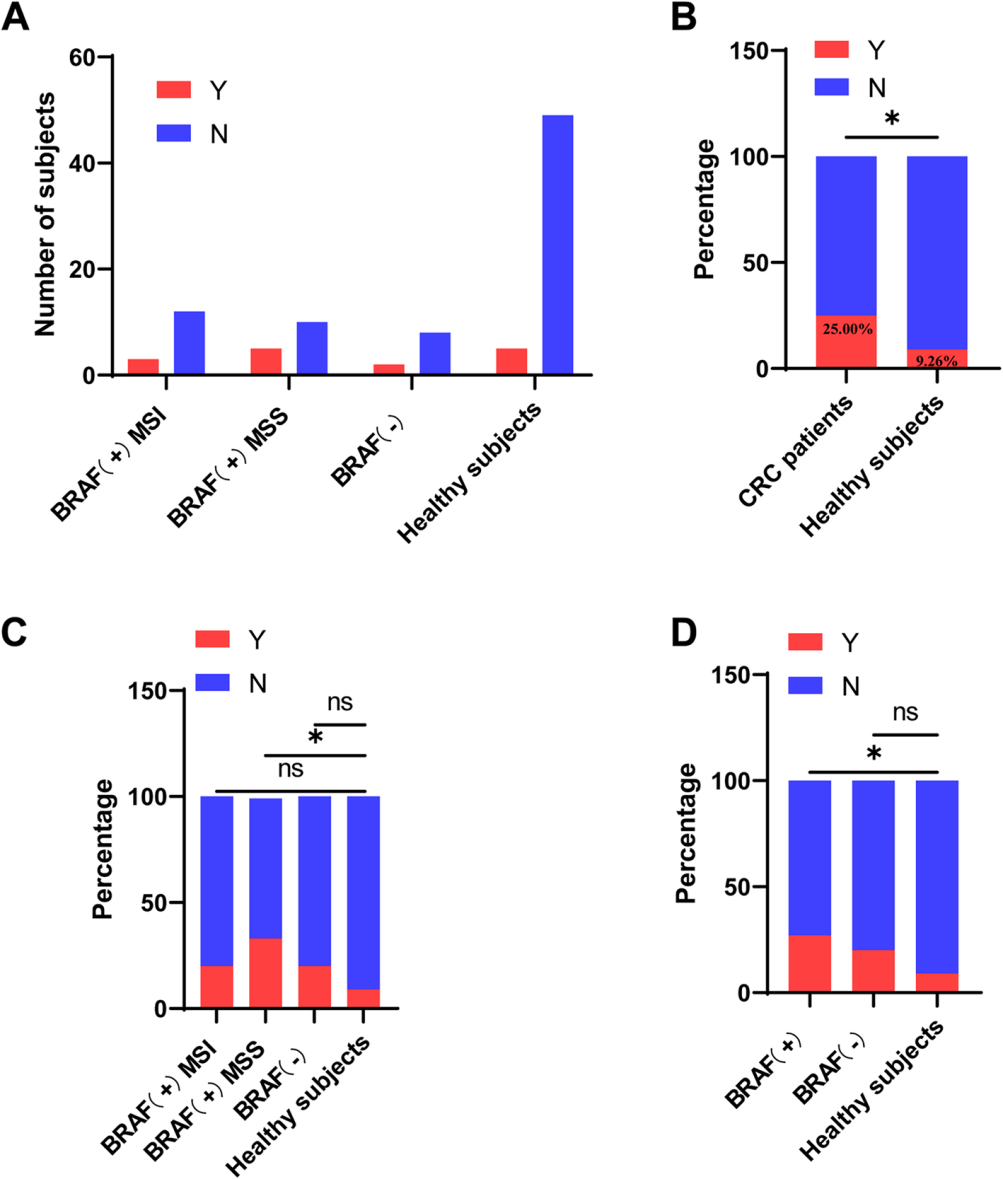
**A** The number of gall-stone subjects in different groups. **B** The percentage of gall-stone in CRC patients and healthy subjects. **C** The percentage of gall-stone in each group. **D** The percentage of gall-stone in CRC patients with or without BRAF mutation and healthy subjects. *P < 0.05

As shown in Table 4, the gallstone rate in the CRC patients (25.0%) and BRAF-positive MSS group (33.3%) was markedly higher than that in the healthy subjects (9.3%) (Fig. 4A–C). However, no significant differences were found between patients in the BRAF-positive MSS group, BRAF-positive MSI group, and the BRAF-wild-type group regarding gallstone (*p* = 0.6412). Moreover, further analysis exhibited that the gallstone rate in the BRAF mutation group (26.7%) was significantly higher than that in the healthy subjects (9.3%) (Fig. 4D).

**Table 4:** BRAF mutation and gallstone in CRC patients and healthy subjects

	Gallstone	
	*Y*	*N*
BRAF + MSI	3	12
BRAF + MSS	5	10
BRAF ( −)	2	8
Healthy subjects	5	49

## Discussion

BRAF, a member of the Raf kinase family, is a cytosolic protein kinase and is activated by membrane-bound RAS. Mutated BRAF activates a signaling pathway, which causes cell proliferation and inhibits apoptosis. The most common mutation is a single glutamic acid to valine substitution at codon 600 causing the V600E point mutation, and the frequency of BRAF mutation is 11% [12]. The BRAF^V600E^ mutation in CRC was associated with advanced TNM stage, poor differentiation, mucinous histology, MSI, and CIMP. This mutation was also associated with female gender, older age, proximal colon, and MLH1 methylation [13]. In BRAF-mutated human CRC cell lines and tumors, MAFG is bound to the promoters of MLH1 and other CIMP genes and recruits a corepressor complex that includes its heterodimeric partner BACH1, the chromatin remodeling factor CHD8, and the DNA methyltransferase DNMT3B, resulting in hypermethylation and transcriptional silencing [14].

There are two types of serrated polyps from which BRAF-mutated cancers originate. The most common one is the sessile serrated adenoma, which occurs predominantly in the proximal colon in old women [15]. For some unknown reason, 25% of them did not have the silenced MLH1 gene and eventually developed BRAF-mutated MSS cancer [2]. In this study, it was also found that the majority of BRAF-mutated CRC was located on the right side of the colon, whereas BRAF MSS CRC accounted for half of the left and right sides, probably due to the size of the samples studied. Although the presence of a BRAF mutation had no prognostic effect in MSI-H cancer, it was strongly associated with a poorer prognosis in MSS cancer [3]. This is consistent with our findings that there was an association between BRAF MSS bowel cancer and its late-stage diagnosis. The accumulation of genetic abnormalities in these cancers also occurs via CIMP, with methylation of different gene promoters rather than in MSI-H cancers, and the silencing of p16 and Wnt pathway genes has been postulated. Mutation of TP53 is more common than BRAF-mutated/CIMP-H/MSI-H carcinomas [16]. Methylation of MGMT, which codes for a DNA repair protein, has also been identified and may be of particular relevance in BRAF mutated/MSS CRC [17]. Although most of TP53 data in this study were incomplete, it can be seen that in the BRAF MSS group, all 3 cases with complete data were TP53 positive, and in the BRAF MSI group, only one of the 6 cases with complete data was TP53 positive. In addition, KRAS are all wild type in the BRAF mutant group, which is consistent with other findings that BRAF and KRAS are mutually exclusive [18]. In the BRAF-wild-type group, 7 out of 10 cases were KRAS mutants, and 4 of them were located on the right side, indicating that KRAS mutants dominated in the right colon, which is one of the reasons why advanced left CRC can benefit from cetuximab and have a better prognosis than advanced right CRC [19].

Why are there two classes of tumors at this site? Factors involved in this bifurcation are currently unknown. Genes that happen to be methylated in colon and other cell lines not only share distinct functional properties (cell signaling, cell adhesion, cell–cell communication, and ion transport) but also have common sequence motifs in their promoters. This suggests that de novo methylation is not a random process but occurs through a specific instructive mechanism [20]. The origins of the colon from the embryonic midgut and hindgut may provide an explanation [1]. Several factors may also contribute to the abnormal hypermethylation, such as exogenous carcinogens, generation of reactive oxygen species, and host genetic differences. The level of DNA methylation is affected by environmental factors. Smoking and chronic inflammation increase DNA methylation [21, 22]. COMT genes catalyze the methylation of various endobiotic and xenobiotic substances preventing quinine formation and redox cycling, which might protect DNA from oxidative damage. A significant association was found between COMT polymorphism (homozygous variant) and P16 methylation [23]. Another potential difference is the impressively increased risk of a positive family history of colorectal cancer associated with the BRAF V600E mutation in microsatellite-stable cancers, suggesting that future exploration of the genetic and/or environmental factors which relate to this association may be fruitful [24].

Gastric cancer (GC) is classified as EBV-positive and MSI-high GC in TCGA [5]. EBVaGC and MSI-GC encompass similar epigenetic traits, including high levels of DNA methylation in CpG islands; however, EBV-positive, and MSI-high GC are mutually exclusive. Sporadic MSI-high GCs are attributable to their MSI with hypermethylation of the promoter CpG island locus and the associated inactivation of the MLH1 gene [25]. All EBV-positive tumors displayed CDKN2A (p16, INK4A) promoter hypermethylation but lacked the MLH1 hypermethylation characteristic of MSI-associated CIMP [5]. The methylation phenotypes of MSS CRC with BRAF^V600E^ mutation and EBVaGC are highly similar. Is EBV infection one of the etiologies of MSS CRC with BRAF^V600E^ mutation? The role of EBV in the pathogenesis of colon lymphoma and other sites such as GC is well established, but its role in CRC is still unclear [26]. EBV has been found to be associated with CRC, but not with CpG island methylation (including MLH1 and P16) [27]. In the tumor stroma, there may be more EBV in tumor-infiltrating lymphocytes (TILs) than in tumor cells [27]. Other studies found that only TILs were EBV positive in CRC samples. Since the design of the analyzed studies, the sample size, and the methodology used for EBV detection varied markedly, they may not lead to meaningful conclusions [26]. The current study cannot determine the effect of EBV on the microsatellite status of BRAF^V600E^ mutant CRC. To this end, we took BRAF^V600E^ mutant CRC as the research object, with microsatellite status as the grouping standard; EBV gold standard EBER assay was used to detect EBV products in tissues. The results suggested that although EBV products were detected in CRC, they were not correlated with MSI status.

GD has been proposed to increase the risk of CRC [28]. It is especially noteworthy that many of the risk factors for GD include factors that are well-established as risk factors for CRC (obesity, high-energy intake, alcohol consumption, and diabetes) [8]. A high consumption of fat and meat, typically characteristics of the diet in western countries, is positively associated with the risk of CRC and linked to comprehensive shifts in gut microbial co-metabolism, including bile acids [29]. This study also found a high correlation between GD and CRC. Although no significant differences were found between patients in the BRAF-positive MSS group, BRAF-positive MSI group, and the BRAF-wild-type group regarding GD, the frequency of GD in CRC reached 25% (10/40), which was higher than the frequency of GD in the general population. Among them, MSS CRC with BRAF^V600E^ mutation group had the highest frequency of GD (33%). A necropsy analysis found that there was a positive association between gallstones and CRC in females only, particularly for right-sided CRC (odds ratio 6.79, 95% *CI* 1.14–46.46) [30].

Bile acids (BAs), particularly secondary BAs, can cause oxidative stress, DNA damage, apoptosis, and mutation and were speculated to be strong carcinogens or promoters of CRC [9, 10]. Downregulation of the farnesoid X receptor promotes colorectal tumorigenesis by facilitating enterotoxigenic *Bacteroides fragilis* colonization [31]. Although the detection of BAs was not performed in this study, in another study reported by our team, deoxycholic acid (DCA) level in the bile of patients with GD was significantly higher than that of the control group, and the expression of FXR in the liver was lower than that of the control group [32]. This is consistent with the findings in CRC, indirectly indicating a high correlation between GD and CRC. DCA acts as FXR antagonist under conditions of genetic instability (e.g., APC loss, β-catenin accumulation), inhibiting FXR function in intestinal epithelial cells and resulting in enhanced WNT signaling and cell cycle progression [33].

This study found an association between GD and BRAF^V600E^ mutant CRC, although there was no significant difference. The relationship, if any, between BAs and consensus molecular subtypes of CRC remains to be explored. The concentration of bile salts higher in the proximal colon and the bile acid profiles was hypothesized to be linked to the increased risk of proximal cancer [34]. When exposed a longer time to dietary DCA, 83% of mice developed sessile adenomas in the proximal colon at 10 months [35]. The changes that occur in the gut-microbiota-liver axis may be of particular interest to proximal colon cancer [36, 37].

The primary cause of CIMP remains unclear. Although they are irrefutably correlated, no mechanistic relationship between CIMP-H and BRAF mutations has been established [16]. Whether BRAF mutation predisposes genes to hypermethylation or hypermethylation and silencing of an unknown gene or gene leads to BRAF mutation or simply that a synergy exists between BRAF mutation and DNA methylation is unclear [11]. Considering the small number of samples in this study, the relationship between EBV and GD and BRAF mutated CRC still needs to be confirmed in a large sample study. However, in patients with gallstone-associated colon cancer, any association with consensus molecular subtypes (BRAF mutated) should be explored to identify if specific pathways are involved [38]. Given the association between GD and BRAF mutation in CRC, the role of environmental factors, especially BAs, in methylation is also worth studying.

As a preliminary study, we acknowledge that there are some unavoidable defects in our research. One of the limitations in this study is the small sample size, which might limit the universality of the results. This partly results from the low incidence of CRCs with BRAF^V600E^ mutation, which accounts for about 10% of CRC patients [12, 39]. More convincing conclusions have yet to be confirmed by further validation in a larger sample. We should not only collect more samples in our hospital but also collaborate with other hospitals and conduct multicenter studies.

The mechanisms of two different microsatellite states in CRCs with BRAF^V600E^ mutation, a topic that is a worldwide challenge, are still inconclusive [40]. This is the purpose of this study, to explore the clue of potential mechanisms from intestinal environmental factors. Indeed, we acknowledge that our existing results are far from revealing the specific mechanisms. In the next step of future researches, various experimental techniques in molecular biology are essential. According to our results, we design to investigate the effect of DCA on BRAF^V600E^ mutation in CRC cells in vitro and in vivo. However, EBV was detected in a very low percentage of our BRAF mutant CRC samples, and there was no difference in distribution. In our opinion, study of the role of EBV in CRCs with BRAF^V600E^ mutation and further RT-PCR or WB of EBV products is not necessary.

The combination of bioinformatic analysis and biological experimental results is the trend of researches in the last several years. We have found a cancer-promoting axis in CRC through this way [41]. Rational application of bioinformatics analysis can help us to screen more promising research directions. In our future research program, KEGG database is used to explore the underlying correlation of BRAF mutation with different microsatellite states and microsatellite loci, and WB analysis of samples is applied to verify the results of bioinformatics analysis. As an advanced and powerful technology, single-cell sequencing is well suited for studies about molecular signaling pathway mechanisms of cancer [42]. In a recent phase 2 trial, single-cell RNA sequencing was applied to confirm a potential mechanism underlying the cooperativity observed between BRAF/MAPK inhibition and immune response [43]. Nevertheless, there are few single-cell sequencing studies on mechanisms of two different microsatellite states in CRCs with BRAF^V600E^ mutation. In consideration of the cost of single-cell sequencing, we offer some suggestions for possible future studies based on our experiences [42]. Before sequencing, the experimental scheme should be well designed, for instance, collecting samples from surgical specimens of BRAF^V600E^ mutation CRC patients with different microsatellite states or from animal tumor models with different treatment groups. Furthermore, experienced bioinformatic analysis technicians contribute to obtaining targeted results, in view of the numerous data generated after sequencing. Finally, in order to identify the authenticity of data from bioinformatic analysis in a biological context, functional validation is an indispensable step [44]. 

In conclusion, this study explored the intestinal environmental factors among different microsatellite status in CRC with BRAF^V600E^ mutation. Although the generalizability of some results is limited, on account of the sample size, we still provide promising directions for studies in this field. Inspiringly, with the applications of single-cell sequencing technologies and the combination of bioinformatic analysis and biological experiments, it is undoubtedly reasonable to foresee that our understanding about the mechanisms of two different microsatellite states in CRCs with BRAF^V600E^ mutation will be significantly advanced in the future.

## Data Availability

All data generated or analyzed during this study are included in this published article and available from the corresponding authors on request.
